# Massachusetts Veterinary Association

**Published:** 1896-05

**Authors:** Howard P. Rogers

**Affiliations:** Secretary


### MASSACHUSETTS VETERINARY ASSOCIATION.

THE regular meeting was held at 19 Boylston Place, Wednesday, February 26th, at 8 P.M., Dr. Parker, the President, in the chair. The following members were present: Drs. Beckett, Blackwood, Bunker, Burr, Cronan, Emerson, Hamilton, Lewis, La Baw, McLaughlin, Osgood, Parker, Pierce, Rogers, Sheldon, Winslow, and Winchester. Honorary member, Dr. Stickney. Minutes of last meeting were read and accepted after Dr. Burr had corrected his statement in regard to tuberculin, having it read "good" instead of "reliable."

The Secretary called the attention of the Association to the fact that the election of Drs. Frothingham and Cannon was illegal, not having been before the Association the length of time prescribed by the Constitution. Dr. Osgood then moved that we again ballot on these names. Carried, and they were unanimously elected.

Dr. Pierce reported the case of a mare six weeks in foal having dizzy spells when first starting on a drive; would have only one attack. After removing overdraw check from harness there was no more trouble.

Dr. McLaughlin reported an interesting case, the history of which is well known. Cow calved and cleaned all right over a year ago, following which she had a number of attacks of supposed indigestion. Last calf came two weeks before time, dead. Inside of one week showed dropsy of legs and abdomen. Diagnosed foreign body in heart. Autopsy showed wire three inches long through right auricle at apex. Abscess at point of entrance of wire. Dr. Burr saw in a post-mortem case the track of a wire from the second stomach to the pericardium; fluid and the wire in the pericardial sac. Dr. Emerson saw a cow that had been fed on lawn-grass; had a number of attacks of supposed indigestion; finally died, and a nail was found in the pericardium.

Dr. Beckett had a large horse with peculiar, sudden lameness; would point and rest on outer toe. No soreness in foot or at any point. Diagnosed possible paralysis. Dr. Burr reported a similar case to that of Dr. Bryden. Dr. La Baw had a similar case. Horse had been cast; on attempting to rise, failed; rolled him over and he got up, but was unable to support body on that leg.

Drs. Bunker and Parker reported undesirable effect from calomel: swelling of lips and tongue and profuse salivation.

HOWARD P. ROGERS, M.D.V.,
Secretary.

